# *Dermacentor marginatus* and *Dermacentor reticulatus*, and Their Infection by SFG Rickettsiae and *Francisella*-Like Endosymbionts, in Mountain and Periurban Habitats of Northwestern Italy

**DOI:** 10.3390/vetsci7040157

**Published:** 2020-10-16

**Authors:** Aitor Garcia-Vozmediano, Giorgia Giglio, Elisa Ramassa, Fabrizio Nobili, Luca Rossi, Laura Tomassone

**Affiliations:** 1 Department of Veterinary Sciences, University of Turin, L.go Braccini, 2, 10095 Grugliasco, Italy; giorgia.giglio@edu.unito.it (G.G.); luca.rossi@unito.it (L.R.); 2 Ente di gestione delle aree protette delle Alpi Cozie, Via Fransuà Fontan, 1, 10050 Salbertrand, Italy; ramassa@alpicozie.eu; 3 Ente di Gestione delle Aree Protette del Po Torinese, Corso Trieste, 98, 10024 Moncalieri, Italy; fabrizio.nobili@collinatorinese.org

**Keywords:** ticks, *Dermacentor marginatus*, *Dermacentor reticulatus*, SFG rickettsiae, wild boar, Italy

## Abstract

We investigated the distribution of *Dermacentor* spp. and their infection by zoonotic bacteria causing SENLAT (scalp eschar neck lymphadenopathy) in Turin province, northwestern Italy. We collected ticks in a mountain and in a periurban park, from vegetation and different animal sources, and we sampled tissues from wild boar. *Dermacentor marginatus* (*n* = 121) was collected in both study areas, on vegetation, humans, and animals, while *D. reticulatus* (*n* = 13) was exclusively collected on wild boar from the periurban area. *Rickettsia slovaca* and *Candidatus* Rickettsia rioja infected 53.1% of the ticks, and *R. slovaca* was also identified in 11.3% of wild boar tissues. *Bartonella* spp. and *Francisella tularensis* were not detected, however, *Francisella*-like endosymbionts infected both tick species (9.2%). Our findings provide new insights on the current distribution of *Dermacentor* spp. and their infection with a spotted-fever group rickettsiae in the Alps region. Wild boar seem to play a major role in their eco-epidemiology and dispersion in the study area. Although further studies are needed to assess the burden of rickettsial diseases, our results highlight the risk of contracting SENLAT infection through *Dermacentor* spp. bites in the region.

## 1. Introduction

In the last few decades, human-induced changes in climate and land use have been favouring the geographic expansion of hard ticks, blood-feeding ectoparasites transmitting microorganisms of remarkable medical and veterinary importance [1]. In addition to *Ixodes ricinus*, the most widespread tick species and well-known disease vector in Europe [2], ticks of the genus *Dermacentor* have increasingly gained attention, both for their geographical spread and their vectorial role. In particular, *Dermacentor reticulatus* has been showing an intensive spread in areas of north-western, north-eastern, and central Europe [3,4,5,6,7,8]. Although the Alps region were considered a barrier for its southern spread [9], recent studies recorded *D. reticulatus* in the northern regions of Italy [10,11,12]. *Dermacentor marginatus* is, conversely, commonly distributed in the Mediterranean basin, including almost the entire Italian territory [13]. These three-host tick species parasitize a wide range of vertebrate hosts, with immatures feeding on small and medium-sized mammals, such as rodents and carnivores, and adult stages preferring larger animals including wild and domesticated ungulates and occasionally humans [7,13,14].

*Dermacentor* spp. are known to transmit a wide range of pathogens, including tick-borne encephalitis (TBE) and Omsk haemorrhagic fever viruses, *Rickettsia* spp., *Francisella tularensis* [15]. Interestingly, Crimean-Congo haemorrhagic fever virus was recently detected in *D. marginatus* in southern Spain [16], reinforcing the hypothesis of its possible involvement in the virus cycle [17]. With regard to the spotted-fever group (SFG) rickettsiae, *Rickettsia slovaca*, *Candidatus* Rickettsia rioja, and *Rickettsia raoultii* have often been reported in *Dermacentor* spp. [18]. These rickettsiae, together with other agents such as *Bartonella henselae* and *Francisella tularensis*, are the causative agents of scalp eschar neck lymphadenopathy—SENLAT in humans, also called tick-borne lymphadenopathy (TIBOLA) or *Dermacentor*-borne necrosis erythema and lymphadenopathy (DEBONEL) [19].

In Italy, a SENLAT outbreak linked to *D. marginatus* bites occurred in the late 2000 [20], in a rural area of Tuscany rich in wildlife, wild boar in particular. Wild boar (*Sus scrofa*), in fact, play an important role as hosts for adult *D. marginatus*, particularly in Mediterranean regions, and they may also feed adult *D. reticulatus* [21,22]. In addition to acting as a maintenance host for adult *Dermacentor* spp., wild boar possibly contribute to the maintenance of some rickettsia [23].

Spotted-fever group rickettsioses are notifiable diseases in Italy but are likely underdiagnosed and underreported. Most reported cases refer to the Mediterranean spotted fever in southern and insular Italy [24], caused by *Rickettsia conorii* and transmitted by *Rhipicephalus sanguineus*. However, diagnosis in humans is frequently based on the research of antibodies against *Rickettsia* spp. and no specific tests are performed to identify the causative rickettsial agent [25]. In northern Italy, data on rickettsioses are scarce; for example, in Piedmont region, the Regional Service for the Epidemiology of Infectious Diseases (SeREMI) registered only 15 human cases in the last decade (2009–2019; [26]), although SFG rickettsiae are commonly detected in ticks from the region and tick bites are increasingly reported [27].

In this study, we investigated the distribution of *Dermacentor* spp. in the Italian Alpine region, in a mountain and in a periurban natural area of Piedmont, and evaluated their infection by zoonotic tick-borne bacteria causing SENLAT. Finally, given the overabundance of wild boar in the periurban areas of Turin city, we tested tissue biopsies of culled animals for the SFG rickettsiae infection to investigate their possible role in the pathogens’ maintenance.

## 2. Materials and Methods

### 2.1. Study Area

The study was conducted in two natural areas in Turin province, northwestern Italy, differing in environmental characteristics, altitude, and abundance of wild ungulates (Supplementary Materials, Figure S1). The first is a mountain area located in the high Susa Valley, including Alpi Cozie regional park (45°03′ N, 6°54′ E) and surrounding areas belonging to the high Susa Valley hunting district. This Alpine valley is characterized by a xeric climate and abundant wild ungulates populations; details about climate, habitat characteristics, and wildlife composition are described in [27]. The second study site is a periurban area located nearby Turin city, belonging to the Po Torinese natural park (45°06′ N, 7°76′ E), which comprises several natural reserves highly fragmented by the presence of urban centers and crops. Po river and its tributaries shape a stepped landscape, where a great variety of ecosystems occur: marsh vegetation, such as reeds (*Phragmites* spp.), prevails along bank streams, together with alders (*Alnus glutinosa*), willows (*Salix* spp.), and black poplars (*Populus nigra*); in hilly areas, mixed broad-leave woods mainly compose the vegetation canopy, with a prevalence of deciduous oaks and sweet chestnuts (*Castanea sativa*). Wild ungulates are in expansion in the area; roe deer (*Capreolus capreolus*) are still rare, while wild boar (*Sus scrofa*) have become overabundant and are subjected to a management plan in order to contain the population.

### 2.2. Dermacentor spp. Ticks Collection

We carried out monthly collections of questing ticks during the spring-autumn seasons, by the dragging method on the ground vegetation in 100 m transects. In the mountain area, dragging was performed from 2016 to 2019 in 45 sites including open-exposed areas, conifers, and broad-leaves woods, ranging altitudes between 959 and 1884 m above sea level (asl), as described in [27]. Conversely, 11 transects from the periurban area were investigated in 2018 and 2019; they included deciduous woods at an altitude between 212 and 587 m asl.

In parallel, we actively conducted tick collection from hunted wild ungulates through skin inspection. The monitoring activity in the mountain area (October–December, 2017 to 2019) was carried out on a hunted game presented at the check station of the local hunting management unit [27]. Some specimens were also collected on owned dogs and livestock (cattle and horses) with the help of Alpi Cozie Park personnel, and on human patients that were visited at a local emergency unit (Susa hospital).

In the periurban area, wild boar subjected to controlled hunting and trapping by forestry authorities, were immediately inspected after culling (October–March, 2017 to 2020). Tissue biopsies (liver or ear tissue) were taken, wherever possible. Data regarding sex, age, and shooting location were recorded. 

All animals sampled in this study were culled by professional hunters in accordance with the Piedmont Regional Law no. 5 of 19 June, 2018 on the protection of fauna and wildlife management–hunting; a veterinarian inspector was always present when animal carcasses were brought at the check station of the local hunting management unit. No animal was harmed for the purpose of sample acquisition.

### 2.3. Tick-Borne Pathogens Detection

Collected ticks were stored in 70% ethanol at room temperature. By using the stereomicroscope, we classified ticks to the stage and species level [13,16], and measured the tick engorgement index—TEI of feeding ticks (index 2) according to [28]. Four *D. reticulatus* were not subjected to molecular analyses, but kept in our tick collection as reference specimens. Tissue biopsies were collected by using sterile scalpel blades and individually stored under RNAlater^®^ Solution (Life Technologies Ltd., Warrington, UK), at −20 °C until the processing and analysis. All *D. marginatus*, nine *D. reticulatus* and tissues were individually homogenized, except for three adult *D. marginatus* that were analyzed in a pool; these were collected from a wild boar in the periurban area at the very beginning of the study. DNA was extracted by using the DNAzol reagent^®^ (Life Technologies LTD, Warrington, UK), as described in [27]. The quality and quantity of extracted DNA samples were evaluated with a spectrophotometer (Nanodrop™ 2000, Thermo Fisher Scientific).

To detect the SFG rickettsiae infection, we primarily used a quantitative polymerase chain reaction (qPCR) assay specific for the detection of *R. slovaca* in ticks and tissues, as described by [29]. Negative samples were further tested by a conventional PCR assay targeting a fragment of gene coding citrate synthase (*gltA*; [30]), in order to identify SFG rickettsiae different from *R. slovaca*. All *gltA*-positive and qPCR-positive samples were eventually subjected to end-point nested-PCR targeting a fragment of *OmpA* gene [31,32], to obtain the nucleotide sequences of *R. slovaca* and other SFG rickettsiae. The infection by *Bartonella* spp. and *Francisella tularensis*/*Francisella*-like endosymbionts (FLEs) was investigated by PCR amplification of 16S-23S rRNA intergenic region [33] and *tul4* gene (PCR and qPCR) [34], respectively. Positive controls and negative water controls were used on every (q)PCR assay performed in this study.

End-point PCRs positive sample amplicons were purified using the ExoSAP-IT™ PCR Product Clean-up Kit (GE Healthcare Limited, Chalfont, UK) and sent to an external service (BMR Genomics, Padua, Italy) for automatic sequencing.

### 2.4. Statistical Analyses

Data were analyzed by using the R software version 3.6.3 for Windows [35]. Prevalence and 95% exact binomial confidence intervals (CI) of tick infestation in wild ungulates and tick and tissue infection were calculated through a binomial exact test. To evaluate significant differences in tick infestation in wild boar according to the animal age and sex, we applied Pearson’s chi-squared test. For these purposes, we categorized the age variable into three groups: ‘Group 0’ included wild boar piglets under 6 months old; ‘Group 1’ included juvenile individuals from 6 to 18 months old; and ‘Group 2’ included adult individuals over 2 years old. Fisher’s exact test was used to compare the *Rickettsia* spp. infection according to the type of tissue analyzed, animal characteristics (age and sex), and shooting location. For all statistical tests, a *p*-value < 0.05 was considered statistically significant.

### 2.5. Phylogenetic Analyses

All nucleotide sequences were primarily handled by using BioEdit [36]. A multiple sequence alignment was performed by the ClustalW algorithm [37], which computes a distance matrix between each pair of sequences based on sequence pairwise comparisons. For bacterial identification, nucleotide sequences were then compared with reference sequences deposited in GenBank throughout BLAST^®^ (Basic Local Alignment Search Tool). Phylogenetic analyses were conducted by applying the neighbour–joining method in MEGA X [38]. The stability of the trees obtained was estimated by a bootstrap analysis with 1000 replicates. Some representative sequences of the *Rickettsia OmpA* gene and FLEs *tul4* gene were submitted to GenBank.

## 3. Results

### 3.1. Tick Collection

We collected 134 *Dermacentor* spp., namely 121 *D. marginatus* and 13 *D. reticulatus*.

*Dermacentor marginatus* was distributed in both study areas, on various hosts and vegetation (Table 1).

With regard to questing *D. marginatus*, immatures were found during summer (June–September) and prevailed over adults (Pearson’s Chi-squared test, *p* < 0.01), that were mainly sampled during spring (March–May) and early autumn (September–October). In the mountain area, *D. marginatus* (10 larvae, two nymphs and six adults) were collected in six dragging transects (13.3% of investigated sites; 95% CI = 5.1 − 26.8), located between 1014 to 1340 m asl [27]. In addition, we opportunistically collected five more adult *D. marginatus* questing on vegetation in a wet pasture frequented by wild boar at 1600 m asl. In the periurban area, *D. marginatus* (17 larvae, one nymph and one adult male) were collected in four dragging transects (36.4% of investigated sites; 95% CI = 10.9 − 69.2), corresponding to recreational hilly areas at altitudes between 278 and 587 m asl. We did not collect *D. reticulatus* on the vegetation in our study areas.

We gathered 92 feeding *Dermacentor* spp. from different animal sources, including wild ungulates, domestic animals, livestock, and humans (Table 1), namely 91 adults and one single nymph collected from a 4-year-old chamois. All feeding ticks had TEI > 2, and TEI was generally much higher in females compared with males.

In the mountain area, we inspected 373 carcasses of hunted wild ungulates [27] and a further moribund male roe deer found by a local veterinary officer. *Dermacentor marginatus* (*n* = 11) infested 6/207 red deer (*Cervus elaphus*; mean tick number per animal: 1.5, min–max = 1–3), 1/24 roe deer (one tick), and 2/143 of chamois (*Rupicapra rupicapra*; one tick per animal). Parasitized animals were culled at altitudes between 1000 and 1700 m asl. Hunters and forestry workers provided further 14 *D. marginatus* from two wild boar (mean tick number per animal: 7, min–max = 4–10), culled at 1600 m asl. Moreover, local farmers collected 10 more *D. marginatus* from dogs and livestock (Table 1) at an altitude of around 1700 m asl. Physicians of the Susa hospital emergency unit also provided two *D. marginatus* adults (one male and one female, in April–May 2019) feeding on two human patients residing in the valley; no data on the clinical status of the patients were provided.

In the periurban area, we exclusively collected feeding ticks from wild boar (Table 1): we visually inspected 102 culled animals and collected 53 adult ticks from 16 carcasses (infestation prevalence 15.7%, 95% CI = 9.2 − 24.2; mean tick number per animal: 3.3, min–max = 1–15). *Dermacentor marginatus* (20 females and 20 males) infested 12.7% (*n* = 13; 95% CI = 7.0 − 20.8) of wild boar (mean tick number/animal: 3.1, min–max = 1–14), while *D. reticulatus* (four females and nine males) parasitized 7.8% (*n* = 8; 95% CI = 3.4 − 14.9) of the animals (mean tick number/animal: 1.6, min–max = 1–5). The two *Dermacentor* species overlapped in time, especially during early spring (March-April) and autumn (November) when five individuals from different localities were found co-infested by both tick species. Tick infestation did not differ among animals according to age (Fisher’s exact test; *p* = 0.103), and sex (Pearson’s Chi squared test; *p* = 0.231). We biopsied liver (*n* = 18) and ear tissues (*n* = 62) from 80 wild boar, of which seven (8.8%; 95% CI = 3.6 − 17.2) were infested by ticks.

### 3.2. Tick-Borne Pathogen Infection

The spotted-fever group rickettsiae infected 53.1% (*n* = 68; 95% CI = 44.1 − 62.0) of *Dermacentor* spp. individually screened, and the pool of three *D. marginatus* collected from wild boar. Tick infection was similar in mountain and periurban areas (Pearson’s Chi squared test, *p* > 0.05), with an overall prevalence of 56.5% (95% CI = 43.3 − 69.0) and 50.0% (95% CI = 37.4 − 62.6) in individual ticks, respectively.

In the mountain area, the prevalence of SFG rickettsiae did not show significant differences between questing and feeding ticks (*p* > 0.05), although this latter group exhibited a greater infection prevalence (59.0% versus 52.2% of questing ticks). On the other hand, the infection prevalence significantly differed when considering the source of ticks in the periurban area (*p* < 0.01), where feeding ticks showed a rickettsial infection prevalence of 60.5% (95% CI = 49.3 − 70.8) versus 21.1% (95% CI = 6.1 – 45.6) in questing ticks.

With regard to the tick life stage, adult ticks (59.8%; 95% CI = 49.3 − 69.6) and nymphs (50.0%; 95% CI = 6.8 − 93.2) were more likely infected compared with larvae (29.6%; 95% CI= 13.8 − 50.2; *p* < 0.05).

Rickettsiae-infected feeding ticks were collected on all animal species, except for horses (Table 1). *Dermacentor* spp. ticks from wild boar showed high infection rates in both study areas (Table 1), with an overall prevalence of 61.5% (95% CI = 47.0 − 74.7) in *D. marginatus* and 55.6% (95% CI = 21.2 − 86.3) in *D. reticulatus*. We likewise recorded a high infection prevalence (75%, 95% CI = 42.8 − 94.5) in *D. marginatus* collected on wild ungulates from the mountain areas, in ticks from deer and chamois. The two *D. marginatus* from human patients also tested positive (Table 1).

Through molecular analyses (qPCR) and nucleotide sequencing, we identified *R. slovaca* in 75.0% (95% CI = 63.0 − 84.7) *Dermacentor* positive ticks, followed by the novel uncultured *Ca*. R. rioja (11.8%; 95% CI = 5.2 − 21.9). However, we failed to determine the rickettsiae species in eight PCR-amplicons given the poor quality of the resulting sequences. We recorded both SFG rickettsiae in questing and feeding ticks in the mountain and periurban areas (Table 1). *Rickettsia slovaca* infected *D. marginatus* from different sources, including questing adults (GenBank accession numbers: MT025712) and nymphs (MT330429), feeding ticks collected from wild ungulates (MT330430-3), and human patients (MT899421). In the periurban area, *R. slovaca* infected *D. reticulatus* ticks as well, in particular a female and two males collected from wild boar. By contrast, *Ca*. R. rioja infected only *D. marginatus*, including questing ticks (MT330435) and feeding ticks from wild boar (MT330436) in both study areas.

Overall, 11.3% (95% CI = 5.3 − 20.3) of the 80 wild boar tissues tested positive for SFG rickettsiae (*gltA* and qPCR). We identified *R. slovaca* in nine tissues, one liver and eight ear biopsies (GenBank accession numbers: MT330434). Two positive ear biopsies belonged to the infested wild boar, one parasitized by a *D. marginatus* male and one by a *D. reticulatus* male. No significant differences were observed in tissue infection according to the animal age, sex, and shooting location (Fisher’s exact test, *p* > 0.05).

**Figure 1:**
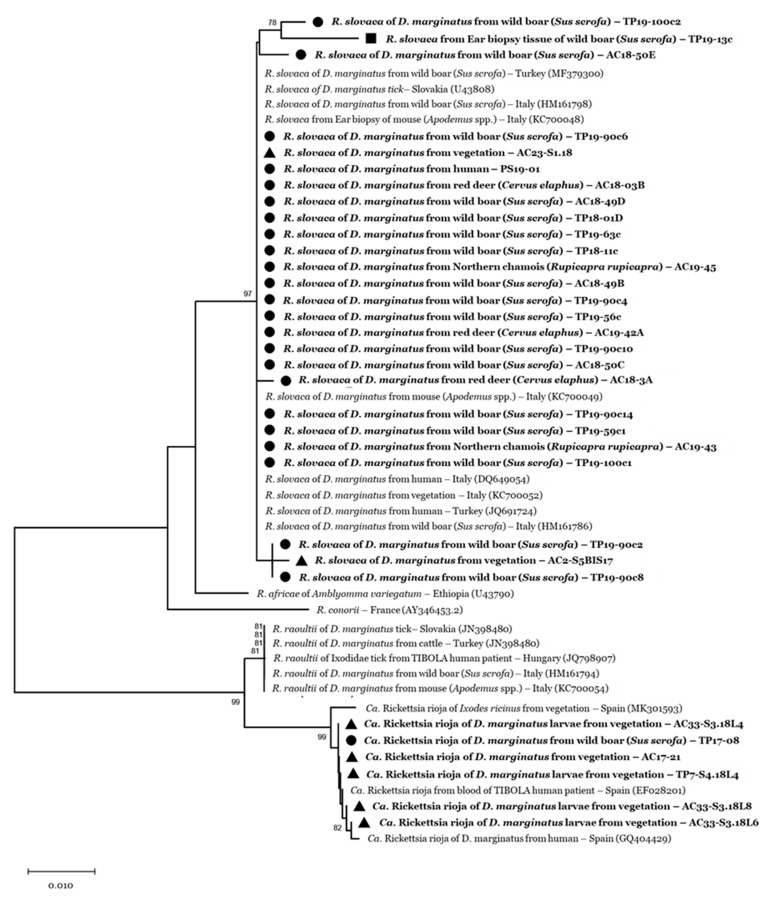
Phylogenetic tree of *OmpA* gene of *Rickettsia* spp. obtained from 32 nucleotide sequences (426 bp) from *D. marginatus* and *D. reticulatus* ticks collected in the study areas (AC: Alpi Cozie regional park; TP: Po Torinese natural park). Reference sequences are identified by the GenBank accession number enclosed in parentheses. Bootstrap values (1000 replications) above 70 are shown next to the internal nodes. Amplicons obtained in this study are indicated with a black symbol: ● Feeding ticks from wild boar, red deer, Northern chamois, and humans; ▲ *Dermacentor* ticks collected from the vegetation; and ■ Ear biopsy collected from wild boar.

Sequences of about 550 bp of the *OmpA* gene of *R. slovaca* showed 100% similarity with sequences identified in *D. marginatus* ticks from other Italian regions (MH532250-7, HM161786-8) and Turkey (MF379300-3-5-11). The sequences identified as *Ca*. R. rioja shared 99–100% identity to sequences of *Ca*. R. rioja (*OmpA* gene) detected in Spain—in the blood of SENLAT-human patient (EF028201), in a *D. marginatus* feeding on a SENLAT human patient (GQ404429), and in questing *I. ricinus* ticks (MK301593-4-5). The *R. slovaca* sequence obtained from our wild boar ear biopsy (GenBank accession number: MT330434), showed 98.6% similarity with the amplified *OmpA* gene detected in questing *D. marginatus* from Turkey (MK922644-53) (Figure 1).

**Figure 2:**
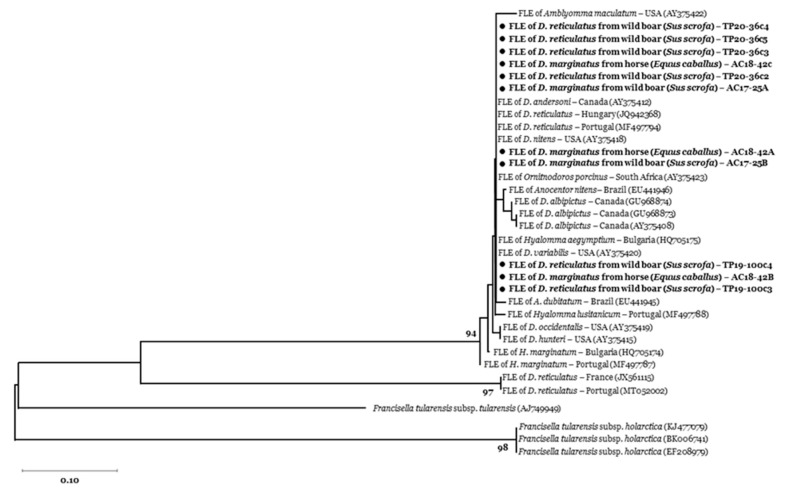
Phylogenetic tree of *tul4* (17 kDa lipoprotein) gene of *Francisella* spp. obtained from 11 nucleotide sequences (200 bp) identified in feeding *D. marginatus* and *D. reticulatus* ticks, respectively collected from horse and wild boar in our study areas (AC: Alpi Cozie regional park; TP: Po Torinese natural park). Reference sequences are identified by GenBank accession number enclosed in parentheses. Bootstrap values (1000 replications) above 70 are shown next to the internal nodes. Amplicons from this study are indicated with a black circle (●).

We did not detect *Bartonella* spp. in our tick sample. *Francisella tularensis* was also absent, as confirmed by the specific qPCR targeting *tul4* gene [34], however 9.2% (95% CI = 4.8 − 15.5) of the ticks tested positive for the *tul4* gene end-point PCR. These positive samples were five *D. marginatus* from the mountain area, collected on a wild boar (*n* = 2) and a horse (*n* = 3), and seven *D. reticulatus* collected from three wild boar from the periurban area (GenBank accession numbers: MT899422-25). Nucleotide sequencing showed 98–100% similarity of our amplicons to *Francisella*-like endosymbionts identified in *D. reticulatus* from Portugal (MF497789-94) and Hungary (JQ942368) (Figure 2).

## 4. Discussion

Our results confirm the widespread distribution of *D. marginatus* and the local presence of *D. reticulatus* in Northwestern Italy. Interestingly, the two species overlap in periurban areas inhabited by wild boar. Moreover, we showed *Dermacentor* spp. infection by two SFG rickettsiae causing SENLAT in humans, *R. slovaca* and *Ca*. R. rioja, and by *Francisella*-like endosymbionts.

The finding of *D. reticulatus* feeding on wild boars was somewhat unexpected. This tick species is indeed widely distributed in central Europe [7,9], but its presence in Italy has been hitherto considered occasional [39,40]. In the Piedmont region, only Maurelli et al. had previously reported some specimens feeding on owned dogs [12]. However, in the bordering Lombardy region, *D. reticulatus* was recently reported in parks located in highly urbanized areas and their presence was linked to canine babesiosis cases [10,11]. Similarly, we detected *D. reticulatus* in the periurban park though not in the mountain study area. According to Estrada-Peña et al., this species prefers urban biotopes and is considered absent in high mountain regions [15]. However, its observation in warm Alpine valleys in France and Switzerland [7] indicate the need of monitoring its presence in the Italian Alpine areas such as Susa Valley, which are experiencing a rise in temperatures [41] and an increase in tick populations [27]. *Dermacentor reticulatus* were collected from wild boar mainly during spring and autumn, which is in line with the period of adults questing activity, also observed in our latitudes by [11]. The occurrence of *D. reticulatus* in close proximity to human settlements entails a potential risk of tick-borne diseases in humans and animals, since this species has a generalist feeding behaviour and is considered a competent vector of *Babesia canis*, SFG rickettsiae (*R. slovaca* and *R. raoultii*), *Francisella tularensis*, but also of TBE virus [7].

*Dermacentor marginatus* ticks, in contrast, are endemic in the Italian territory [13]. We recorded their occurrence from 200 m up to 1700 m asl, in the vegetation and on animals from mountain and periurban areas. Our results confirm the great plasticity of *D. marginatus* and adaptability to different environments. For example, Selmi et al. reported *D. marginatus* in different habitats (e.g., typical Mediterranean woods, meadows, and croplands), up to 1600 m asl [42]. Questing immature *D. marginatus* were the most common life stages encountered and prevailed during the summer, while the few adult ticks were mainly collected during spring, indicating a potential risk period for *D. marginatus* bites in humans. We unexpectedly analyzed also two *D. marginatus* collected on human patients at the local emergency unit. *Dermacentor* bites are uncommon in Piedmont region [43] and, in general, they are less frequent compared with bites of generalist ticks such as *Ixodes ricinus* [13,14]. This finding may indicate a significant presence of *D. marginatus* in the area, although with a focal distribution [27], and the chance for humans to be bitten.

Spotted-fever group rickettsiae infected around 50% of our *D. marginatus* sample. This prevalence is slightly lower than the infection prevalence observed in central Spain (63.9%) in questing and feeding *D. marginatus* collected from livestock and wild mammals [44]. Regard questing ticks, the SFG rickettsiae infection in our *D. marginatus* (38.1%) is similar to the infection prevalence observed in *D. marginatus* in Spain (35.8%) and Tuscany (36.4%) [45,46].

The overall occurrence of SFG rickettsiae in *Dermacentor* spp. was similar in the mountain and periurban areas. We observed a higher infection rate in feeding ticks compared to questing ticks, although the difference was significant in the periurban area only; this finding is in contrast with previous studies that reported similar *Rickettsia*-infection rates between *Dermacentor* spp. feeding on animals and questing [20,46,47]. In addition, adults and nymphs were significantly more infected than larvae. These results could be possibly due to the chance of ticks getting infected during the blood meal on the vertebrate hosts, either by systemic infection or by co-feeding [48]. Ticks are considered a reservoir for *Rickettsia* spp., thanks to transovarial and transstadial transmission [49]. Whether vertebrate hosts can serve as reservoir of SFG rickettsiae is still under debate [48]. Some studies previously suggested a possible role of small mammals [50] and wild boar [42,45,46] in the transmission cycle of *R. slovaca*. We indeed detected *R. slovaca* in wild boar tissues, with a prevalence of 11.3%, which is comparable with 11.1% of prevalence reported in skin biopsies from wild boar in Tuscany [46]. Our finding of rickettsiae-infected ear tissues suggests, at least, a local infection after the bite of infected ticks, so wild boar might behave as an amplifier host of the pathogen through co-feeding [48]. Nevertheless, if a local infection in the auricular tissues occurred, it persisted for a period time following the tick bite, since none of the wild boar with *R. slovaca*-positive tissues were found infested with ticks at the time of culling. In addition, the detection of *R. slovaca* in a liver sample might suggest a systemic circulation of the pathogen, which is in accordance with the finding of the pathogen in spleen tissues of wild boar from Algeria [51]. Further studies are needed to investigate this hypothesis and clarify whether the rickettsiemia in wild boar reaches a sufficient level for the bacterial transmission to ticks during the blood meals. Anyway, wild boar seems to play a role as maintenance host for *Dermacentor* adults in our study areas, including *D. reticulatus*. Wild boar were indeed the most infested animal species and showed higher tick loads compared to the other wild ungulates. Moreover, wild boar may disperse tick vectors in close proximity to human settlements, thanks to their ability to adapt and exploit even highly anthropized contexts [42]. In fact, we recovered questing *Dermacentor* spp. very close to urban areas in the Po Torinese natural park (Supplementary Materials, Figure S1).

Deer and chamois in the mountain area were also infested by *D. marginatus*, though with lower frequency and infestation burdens compared to *I. ricinus* [27]. Although we may have underestimated tick loads, since we only visually inspected the animal carcasses, our findings suggest a minor role of these ungulate species as maintenance hosts for *D. marginatus* compared to wild boar. Unfortunately, it was not possible to take biopsy samples to investigate their possible infection by *Rickettsia* spp.

*Rickettsia slovaca* was the most common rickettsia infecting our *D. marginatus* ticks (74.3% of mountain and 72.4% of periurban rickettsiae-positive sample). The pathogen infected 47.2% of *D. marginatus* collected from wild boar; this prevalence exceeds the infection rates previously observed in northeastern Spain (30.5%) and central Italy (32.1%) [45,46]. More recent studies, conducted in different regions of the Italian territory, reported a comparable prevalence of *R. slovaca* in *D. marginatus* collected from wild boar in Liguria (40.7%), but also lower infection rates in Sardinia (33.3%) and its absence in Elba Island, Tuscany [23]. Cicculli et al. [52] first recorded *R. slovaca* infecting *D. marginatus* from wild boar in Corsica, with a prevalence significantly lower than our report (15.4%).

Both *D. marginatus* from human patients tested positive for *R. slovaca*, and since both ticks were engorged, the transmission of the pathogen may have occurred; unfortunately, we did not get data on the health status of the patients. Infection by the SENLAT syndrome was reported in several European areas, such as Tuscany in Italy [46], Spain [53], and France [19,54]. In Piedmont region, Dutto and Selmi reported a case of disease in 2012, with symptoms compatible with the SENLAT syndrome, in a woodcutter bitten by *D. marginatus* in the parietal region [55]. This report indicates that the disease may have been present in the region for a long time.

Three out of nine *D. reticulatus* collected from wild boar also tested positive for *R. slovaca*. Previous studies report a *R. slovaca* prevalence of 28.8% in *D. reticulatus* feeding on horses, goats, and dogs in Slovakia [47], and in 25% questing ticks from Spain [56]. *Dermacentor reticulatus* is considered a competent vector for the SENLAT syndrome [19]. Hence, despite the modest presence of this tick vector in our study areas, our results highlight the potential risk for humans to contract the infection through *D. reticulatus* bites in Turin periurban areas.

To our knowledge, we first report *Ca*. Rickettsia rioja in Italy, which was detected in *D. marginatus* from wild boar and vegetation. *Candidatus* R. rioja was first identified in 2006 in Spain, on feeding ticks collected from SENLAT-human patients and subsequently characterized to the molecular level in 2009 [53,57], but it is still uncultured to date. Its pathogenicity has been recognized and the bacterium constitutes, alongside *R. slovaca* and *R. raoultii*, one of the causative agents of SENLAT syndrome [18]. The rickettsia was reported in human patients affected by SENLAT in Spain and France [19,58]. Notwithstanding, the similarity of the nucleotide sequence of *Ca*. R. rioja with that of *R. raoultii* hinders its identification, in particular when the *OmpA* gene is targeted for its amplification [56,57,59]. Upon discovery, *Ca*. R. rioja has been recorded in both feeding and questing ticks, including *D. marginatus* collected from a woman patient affected by SENLAT [58], but also in *D. marginatus*, *D. reticulatus,* and *I. ricinus* collected from the vegetation [56]. Given its recent discovery and the difficulty for its identification, it is conceivable that the prevalence and spread of *Ca*. R. rioja are underestimated in the literature. The prevalence of *Ca*. R. rioja (6.2%) in our study was significantly lower than that observed for *R. slovaca* (39.5%), however Remesar et al. [56] recorded a similar prevalence of both rickettsiae species in *D. marginatus* collected from the vegetation in northern Spain.

We did not find *Bartonella* spp. in our tick sample. In previous studies, *Bartonella* spp. were detected in 21.4% of *D. reticulatus* in Siberia [60], in 9% of *D. marginatus,* and 12% *D. reticulatus* in France [61], while it was not detected in *D. marginatus* from Sardinia, Italy [62,63]. The possible role of *Dermacentor* spp. as *Bartonella* vector deserves investigations since the *B. henselae* infection in humans was reported following an infected *Dermacentor* spp. bite [64] and is considered a SENLAT agent [19]; moreover, other tick species, such as *I. ricinus*, were recognized as vectors of *Bartonella* spp. [65,66].

We did not record the infection by *F. tularensis* in *Dermacentor* spp., as in other European studies [61,67]. The role of ticks in its transmission is indeed debated [68]. We instead identified FLEs in both *D. marginatus* and *D. reticulatus*. Infection prevalence was below 10%, comparable to studies carried out in Bulgaria [69] and Hungary [70], and lower than infection rates reported in Portugal [71] and France [34]. Studies carried out in northwestern and southern Italy have recently reported the circulation of FLEs in tick species different from *Dermacentor* spp., such as *Hyalomma* spp. and *Rhipicephalus* spp., collected from different animal hosts [63,72]. Occurrence of these maternally inherited symbionts seems to be crucial for tick survival since these bacteria may provide nutritional components, such as B-group vitamins, that normally lack in blood meals [73,74]. Co-speciation of FLEs and *D. reticulatus* has been previously suggested [34], however, our results on FLEs phylogeny do not support this hypothesis and are in line with previous studies that indicate a relatively recent association between the bacterium and *Dermacentor* ticks [75]. Future investigations will need to clarify if FLEs infection in ticks interferes with the prevalence of pathogenic *Francisella* strains, as previously suggested [71].

## 5. Conclusions

In southern Europe, the health threats posed by *Dermacentor* spp. ticks come abreast to those posed by other tick species such as *I. ricinus*, which shows a remarkable geographical expansion [27]. Our findings highlight the circulation of *D. reticulatus* in addition to *D. marginatus* in Piedmont region, and their infection with causative agents of the SENLAT syndrome (*R. slovaca* and *Ca*. Rickettsia rioja). *Dermacentor* spp. showed a wide distribution, from periurban to high mountain habitats, and parasitized a wide range of hosts, including humans. Wild boar, in particular, seem to play a major role in their eco-epidemiology in the study area. Therefore, we can state that northwestern Italy is at risk for SENLAT. A higher notification rate of tick-borne diseases to the health authorities, and the use of routine biomolecular diagnostic tests to confirm rickettsial infections and identify the specific causative agents, would help in assessing the effective burden of rickettsial diseases in the region.

## Figures and Tables

**Table 1:** *Dermacentor* spp. ticks by geographic location and host source and their infection by the spotted-fever group (SFG) rickettsiae; Turin province, 2016–2020.

Location	Source	Tick Species	N Ticks Tested	% SFG Rickettsiae Infection [95% CI]	
				*R. slovaca*	*Ca.* R. rioja
**Mountain area**	Vegetation	*D. marginatus*	23	27.3 [10.7–50.2]	4.5 [0.1–22.8]
	Chamois		2	50 [1.3–98.7]	0
	Red deer		9	77.8 [40.0–97.2]	0
	Roe deer		1	100 [2.5–100]	0
	Wild boar		14	35.7 [12.8–64.9]	7.1 [0.2–33.9]
	Cattle		4	50.0 [6.8–93.2]	0
	Dog		3	33.3 [0.8–90.6]	0
	Horse		3	0	0
	Human		2	100 [15.8–100.0]	0
	nd		1	100 [2.5–100]	0
**Periurban area**	Vegetation	*D. marginatus*	19	10.5 [1.3–33.1]	5.3 [0.1–26.0]
	Wild boar	*D. marginatus*	40	52.6 [35.8–69.0]	5.3 [0.6–17.7] *
		*D. reticulatus*	9	33.3 [7.5–70.1]	0

nd: Engorged female of *D. marginatus* from unknown animal source; * Minimum infection rate, including individually tested ticks and the pool of three adults of *D. marginatus.*

## Supplementary Materials

The following is available online at https://www.mdpi.com/2306-7381/7/4/157/s1. Figure S1: Study areas in Turin province: High Susa Valley (mountain; (**A**) and Po Torinese natural park (periurban area; (**B**). Maps illustrate dragging transects, land use characteristics, and the presence of questing *Dermacentor marginatus* in each location during the dragging sampling period (2016–2019).
